# Clinicopathological Features and Prognoses in Patients With Brain Metastases From Small-Cell Esophageal Carcinoma: A Retrospective Analysis of 18 Patients

**DOI:** 10.3389/fonc.2021.654727

**Published:** 2021-04-16

**Authors:** Wenpeng Jiao, Chi Lin, Linlin Xiao, Xinyuan Zhang, Mengzhu Hu, Min Zhao, Jun Wang

**Affiliations:** ^1^Department of Radiation Oncology, The Fourth Hospital of Hebei Medical University, Shijiazhuang, China; ^2^Department of Radiation Oncology, University of Nebraska Medical Center, Omaha, NE, United States; ^3^Department of Oncology, Hebei Chest Hospital, Shijiazhuang, China

**Keywords:** small-cell esophageal carcinoma, brain metastases, brain radiotherapy, prognosis, ds-GPA

## Abstract

**Objective:** This study aims to examine the clinical characteristics of patients with brain metastases (BM) from small-cell esophageal carcinoma (SCEC) and to explore the association of the corresponding factors with overall survival (OS).

**Methods:** The data of 18 patients with brain metastases from SCEC, diagnosed from January 1, 2006, to December 31, 2018, in the Fourth Hospital of Hebei Medical University were analyzed retrospectively.

**Results:** The 18 patients who were included in this study accounted for 6.7% of the patients with SCEC diagnosed from 2006 to 2018. Of the 18 patients, 8 (44.4%) were females. For the entire cohort, the median OS was 7 months, the 1-year OS was 22.2%, and the 2-year OS was 0%. For patients who received whole-brain radiotherapy (WBRT) and for those who did not (13 vs. 5), the median OS was 11.9 and 3 months, respectively, and the 1-year OS was 30.8 and 0%, respectively. When comparing diagnosis-specific Graded Prognostic Assessment (DS-GPA) scores of patients with BM from SCEC ranging from 2.5 to 4 and from 0 to 2, the median OS was 13.1 and 4 months, respectively, and the 1-year OS was 57.1 and 0%, respectively. In the univariable regression, patients who received WBRT had improved OS compared to those who did not (HR = 0.249, *p* = 0.018), and patients with a DS-GPA score of 2.5–4 were associated with improved OS compared with patients with a DS-GPA score of 0–2 (HR = 0.050, *p* = 0.005).

**Conclusion:** The incidence of brain metastases in patients with SCEC is low, but the prognosis in those patients is very poor. The DS-GPA score may be a prognostic factor of patients with BM from SCEC. Brain radiotherapy could improve the survival of these patients.

## Introduction

The esophagus is the most common site for small-cell carcinoma outside the lung (1), which has the characteristics of high malignancy, poor prognosis, and early metastasis. Small-cell lung carcinoma (SCLC) is highly prone to brain metastasis (BM). Although some clinical features of small-cell esophageal carcinoma (SCEC) are similar to that of SCLC (2), the incidence rate of SCEC is very low, accounting for only 0.05 2.40% of esophageal cancer (EC) (3). Because of the low incidence rate of SCEC, studies of BM from SCEC have mostly been found only in case reports. Therefore, the risk factors, the best treatment modalities, and prognosis of the patients with BM from SCEC are largely unknown.

Treatment of BM is influenced by the performance status of the patient; the location, size, and the number of lesions of the metastatic tumors in the brain; and the status of extracranial metastases, among other factors. At present, the prognosis for patients with BM is usually evaluated by the diagnosis-specific Graded Prognostic Assessment (DS-GPA). For patients with a digestive tract tumor, the prognosis can be graded according to the Karnofsky Performance Status (KPS), age, the number of BM lesions, and the status of extracranial metastases. For BM, the main treatment modalities include surgery, radiotherapy, and systemic therapy. Brain radiotherapy can reduce 80–90% of the neurological symptoms caused by BM (4, 5). Domeki et al. (6) have shown that seven patients who reported with EC and BM received relief from their neurological symptoms after Gamma Knife or whole-brain radiotherapy (WBRT). The median overall survival (OS) was only 3.3–3.9 months for patients with EC and BM, which is worse than that for lung cancer or breast cancer. Feng et al. (7) reported six cases of patients with SCEC and patients with BM, and the median OS for the patients was only 6 months. At present, the clinicopathological factors, treatments, and prognostic factors of BM from SCEC are not clear and are rarely reported in the literature.

The data of 18 patients with BM from SCEC treated from January 2006 to December 2018 at the Fourth Hospital of Hebei Medical University were analyzed retrospectively. From the perspective of the literature review, it was the largest research object of SCEC with BM. The objectives of this study were (1) to investigate the clinicopathological characteristics of BM from SCEC, (2) to explore the outcomes and prognostic factors of BM from SCEC, and (3) to identify effective therapeutic modalities for patients with BM from SCEC, which would help modify clinical management.

## Materials and Methods

### Clinical Data

The data of the patients with SCEC and patients with BM admitted to the Fourth Hospital of Hebei Medical University from January 1, 2006 to December 31, 2018 were analyzed retrospectively. The exclusion criteria included: (1) BM caused by other types of malignant tumor and (2) lack of follow-up information. The primary SCEC lesion was confirmed by histopathological examination. Patients were staged using the TNM system and the BM was diagnosed using histopathological or imaging examinations, such as brain CT, MRI, or PET-CT scans. Clinicopathological data and treatment methods were collected from medical records or *via* telephone follow-ups. The last follow-up was done on June 15, 2020. This study was approved by the Ethics Committee of the Fourth Hospital of Hebei Medical University.

### Endpoint

Overall survival was defined as the time from the date of the diagnosis of BM to the date of death or of the last follow-up.

### Variables

Clinical variables, including gender, age at diagnosis, length of the primary tumor, site of the primary tumor, treatment modalities, number of BM lesions, and the status of extracranial metastases, were analyzed. The univariable analysis included gender, age at diagnosis, number of BM lesions, the status of extracranial metastases, the DS-GPA score, and data from brain radiotherapy and chemotherapy.

### Prognosis Grades

The DS-GPA score is calculated according to the latest DS-GPA standard, which includes KPS, age, number of BM lesions, and the status of extracranial metastases (3). In this study, 11 cases expressed DS-GPA scores of 0–2, and 7 cases expressed DS-GPA scores of 2.5–4.

### Follow-Up

All patients were followed up until June 15, 2020. The follow-up methods were outpatient reexamination, telephone follow-up, and medical record review. On the last date of follow-up, all 18 patients were dead.

### Statistical Method

The SPSS 18.0 software was used for statistical analysis. The Kaplan–Meier curves were plotted to report the OS, and a log-rank test was used to indicate the significance of the findings. The χ2 test or the Fisher's exact test was used for the comparison of rates between the groups. Statistical significance was set at *p* < 0.05, and all tests were two-tailed.

## Results

### Patient Characteristics

Eighteen patients were included in this study, which accounts for 6.7% of the SCEC cases diagnosed between 2006 and 2018. The mean (± SD) and the median (range) time between the diagnosis of SCEC and the development of BM were 11.1 (± 8.4) months and 8.7 (1–37) months, respectively. The median (range) age at diagnosis was 59.5 (46–73) years, including 16 patients aging <70 years old and two patients aging ≥ 70 years. Out of 18 patients, 10 were males. The primary tumors were located in either the mid-thoracic (14 cases) or the low-thoracic (four cases) esophagus. Seven patients had tumors of <5 cm in length, and 11 patients had tumors of ≥5 cm in length. There were seven cases with CD56 positive, seven with Syn positive, four with ChrA positive, three with ChrA weak positive, seven with CK positive, four with NSE positive, two with NSE weak positive, and one with NSE negative. Nine patients had single BM, 14 patients had BM alone, and four patients had synchronous extracranial metastasis, as detailed in Table 1.

**Table 1:** Characteristics of patients.

**Clinical factor**	**Number of cases (%)**
Gender	
Male	10 (55.6%)
Female	8 (44.4%)
Age	
<70 years old	16 (88.9%)
≥70 years old	2 (11.1%)
DS-GPA	
0–2	11 (61.1%)
2.5–4	7 (38.9%)
Lesion length	
<5 cm	7 (38.9%)
≥5 cm	11 (61.1%)
Lesion site	
Middle thoracic esophagus	14 (77.8%)
Lower thoracic esophagus	4 (22.2%)
Initial treatment	
Surgery + chemotherapy	8 (44.4%)
Radiotherapy + chemotherapy	7 (38.9%)
Surgery	1 (5.6%)
Radiotherapy	2 (5.6%)
Brain metastases	
Single	9 (50%)
Multiple	9 (50%)
Extracranial metastases	
Yes	4 (22.2%)
No	14 (77.8%)

### Treatment for BM

Among the 18 patients, four were treated with only WBRT, five were treated with only chemotherapy, and nine were treated with WBRT combined with chemotherapy.

Among the 13 patients who received WBRT, seven patients received WBRT of a dose of 30–40 Gy, six patients received WBRT followed by a boost to the gross tumor of a total dose of 50–60 Gy, two patients received WBRT plus paclitaxel/platinum chemotherapy, six patients received WBRT plus irinotecan/platinum chemotherapy, and one patient received WBRT plus etoposide/platinum chemotherapy.

Among the five patients who received only systemic chemotherapy, two patients received irinotecan plus platinum, two patients received paclitaxel plus platinum, and one patient received etoposide plus platinum. The median chemotherapy cycle was four (ranging 1–6) cycles (Table 2).

**Table 2:** Treatment of 18 patients.

**Patient ID**	**Age**	**Gender**	**DS-GPA**	**Initial treatment stage**	**Initial treatment**	**PFS (to brain metastasis)**	**Numbers of Brain metastasis**	**Combined extracranial metastasis**	**Treatment after brain metastasis**	**OS after brain transfer (Months)**
1	46	Female	2	Limited stage (T1N0M0)	Surgery → EP*4	PFS = 5	Multiple	Liver, bone, lung	Brain radiotherapy + (Irinotecan + Platinum)^*^4	11
2	50	Male	4	Limited stage (T3N0M0)	Surgery → EP*2	PFS = 4	Single	—	Brain radiotherapy + (Irinotecan + Platinum)^*^4	12
3	50	Female	3	Limited stage (T3N0M0)	Surgery → EP*4	PFS = 15	Single	—	Brain Radiotherapy + (Irinotecan + Platinum)^*^4	17
4	51	Female	2	Limited stage	Radiotherapy → EP*3	PFS = 9	Single	—	Taxus + platinum^*^1	1
5	54	Male	3	Limited stage (T4N2M0)	Surgery → EP*4	PFS = 7	Multiple	—	Brain radiotherapy	12
6	57	Male	2.5	Limited stage	Radiotherapy → EP*2	PFS = 14	Single	—	Taxus + platinum^*^4	10
7	58	Female	1	Limited stage (T3N1M0)	Surgery → Etoposide Capsules^*^6	PFS = 12	Multiple	—	Irinotecan + platinum^*^2	2
8	58	Male	0.5	Limited stage (T3N1M0)	Radiotherapy → FLP*4	PFS = 37	Multiple	Right Supraclavicular Lymph Node	Brain radiotherapy + supraclavicular radiotherapy+EP*4	5
9	59	Female	1	Limited stage (T2N0M0)	Surgery → EP*6	PFS = 7	Multiple	—	Brain radiotherapy + (Irinotecan + Platinum)^*^2	2
10	60	Male	1.5	Limited stage	Radiotherapy → FLP*2	PFS = 3	Single	—	EP*5	7
11	61	Male	1	Limited Stage	Radiotherapy → EP*4	PFS = 11	Multiple	—	Brain radiotherapy + (Irinotecan + Platinum)^*^4	6
12	61	Male	2.5	Limited stage (T4N0M0)	Surgery → EP*6	PFS = 7	Single	—	Brain radiotherapy + (Taxanes + Platinum) ^*^6	20
13	64	Male	0.5	Limited stage (T1N0M0)	Surgery	PFS = 8	Multiple	Lymph Nodes on The Right Side of The Neck	Irinotecan + Platinum^*^2	3
14	65	Female	0	Limited stage (T1N0M0)	Surgery → FLP*4	PFS = 5	Multiple	Liver	Brain radiotherapy + (Taxanes + Platinum)^*^1	2
15	65	Male	1	Limited stage	Radiotherapy → EP*1	PFS = 2	Multiple	—	Brain radiotherapy + (Irinotecan + Platinum)^*^4	12
16	67	Female	2.5	Limited stage	Radiotherapy → EP*4	PFS = 7	Single	—	Brain radiotherapy	13
17	70	Male	1.5	Limited stage	Radiotherapy	PFS = 14	Single	—	Brain radiotherapy	4
18	73	Female	2.5	Limited stage	Radiotherapy	PFS = 8	Single	—	Brain radiotherapy	13

* How many cycles of chemotherapy are performed. EP*4 has done 4 cycles of EP regimen chemotherapy.

### Survival

**Figure 1:**
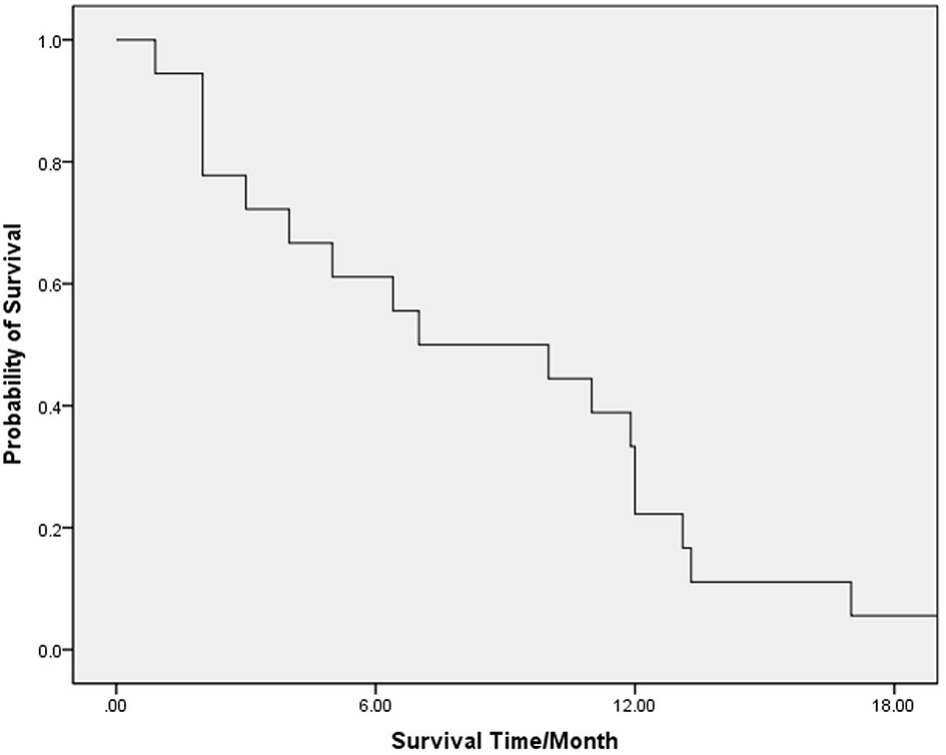
OS for all patients.

The median (range) OS of the whole group of patients was 7 (1–20) months. The 6-, 12-, and 18-month survival rates were 61.1, 22.2, and 5.6%, respectively (Figure 1).

In the univariable Cox proportional hazard analysis (Table 3), patients with DS-GPA scores of 2.5–4 were associated with improved OS as compared to patients with DS-GPA scores of 0–2; patients who received WBRT had improved OS compared to those who did not. On the other hand, the age of, the gender of, the number of BM lesions in, the status of extracranial metastases in, and the receipt of chemotherapy in patients were not associated with OS.

**Table 3:** Univariable cox proportional regression analysis of factors associated with overall survival (OS).

**Clinical factors**	**Number of cases**	**Median survival time (Months)**	**HR**	**95% CI**	***P***
Gender			1.109	0.417~2.953	0.833
Male	10	7			
Female	8	2			
Age			0.993	0.222~4.445	0.993
<70 years old	16	7			
≥70 years old	2	4			
Brain metastasis			1.372	0.520~3.621	0.515
Single	9	10			
Multiple	9	5			
Extra-brain metastasis			2.796	0.798~9.804	0.092
Yes	4	3			
No	14	10			
DS-GPA			0.050	0.006~0.408	0.005
0–2	11	4			
2.5–4	7	13.1			
Radiotherapy			0.249	0.071~0.874	0.018
Yes	13	11.9			
No	5	3			
Chemotherapy			1.185	0.405~3.466	0.752
Yes	14	7			
No	4	12			

**Figure 2:**
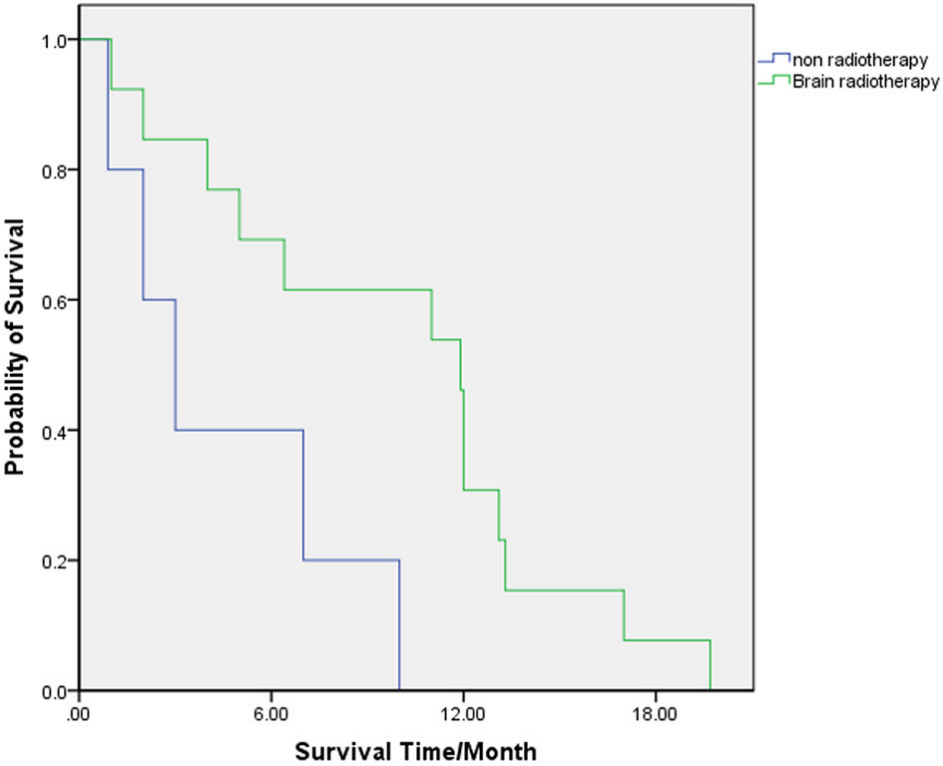
OS for patients who received WBRT (Green) and for who did not (Blue).

The clinical factors of the patients who received WBRT and of who did not were compared, and the results showed no significant difference between the two groups (Table 4). In patients who received WBRT, the median (range) OS was 11.9 (2–20) months. The 6-, 12-, and 18-month-OS rates were 69.2, 30.8, and 7.7%, respectively. In patients who did not receive WBRT, the median (range) OS was 3 (1–10) months. The 6-, 12-, and 18-month-OS rates were 40, 0, and 0%, respectively. There was a significant difference in the risk of death between the two groups (HR = 0.249, 95% CI: 0.071 0.874, *p* = 0.018) (Table 3; Figure 2).

**Table 4:** Comparison of clinical factors between patients who received whole-brain radioteraphy (WBRT) and those who did not.

**Clinical factors**	**WBRT**	**NO WBRT**	***P***
Gender			1.000
Male	7	3	
Female	6	2	
Age			1.000
<70 years old	11	5	
≥70 years old	2	0	
Brain metastasis			1.000
Single	6	3	
Multiple	7	2	
Extra-brain metastasis			1.000
Yes	3	1	
No	10	4	
DS-GPA			0.596
0-2	7	4	
2.5-4	6	1	

**Figure 3:**
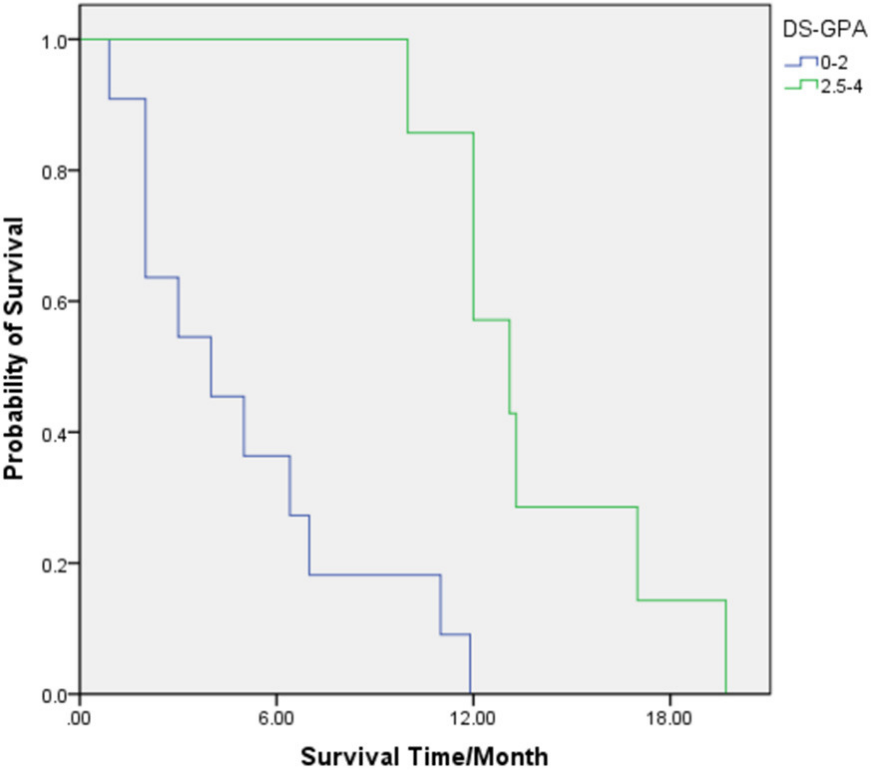
OS for patients with diagnosis-specific Graded Prognostic Assessment (DS-GPA) scores of 0–2 (Green) and 2.5–4 (Blue).

For patients with the DS-GPA score of 0–2, the median (range) OS was 4 (1–11) months, and the 6-, 12-, and 18-month-OS rates were 27.3, 0, and 0%, respectively. The median (range) OS of patients with the DS-GPA score of 2.5–4 was 13.1 (10–20) months. The 6-, 12-, and 18-month-OS rates of these patients were 100, 57.1, and 14.3%, respectively. The prognosis of patients with the DS-GPA score of 2.5–4 was significantly better than that of patients with the DS-GPA score of 0–2 (HR = 0.050, 95% CI: 0.006 0.408, *p* = 0.005) (Table 3; Figure 3).

## Discussion

Small-cell esophageal carcinoma is highly malignant and is easy to metastasize in the early stages. The common metastatic sites are the liver, lungs, bones, and the brain (8). Because of the low incidence rate and high mortality rate of SCEC, studies of BM from SCEC have mostly been found in case reports. To our knowledge, this study has the largest cohort of patients with BM from SCEC. We have shown that the incidence of BM in SCEC is 6.7%, which is much higher than the incidence of BM in esophageal squamous cell carcinoma (ESCC), which is 0.49–1.4% (9, 10). Patients analyzed in this study had a limited-stage disease at diagnosis. The average interval from the initial diagnosis to the occurrence of BM was 11.1 ± 8.4 months. This interval suggested that, for these patients, after completion of the surgery, radiotherapy, and systemic chemotherapy for the primary tumor, monitoring for BM is necessary. Brain screening should be considered in the early follow-up period, especially within 1–2 years after the treatment, to facilitate early detection and early treatment of BM.

The clinicopathological characteristics of SCEC are distinct from ESCC and esophageal adenocarcinoma (EAC) because of the special pathological subtype of SCEC. In the present study, male patients with BM from SCEC accounted for 55.6%, with a 5:4 male-to-female ratio. This male-to-female ratio was significantly lower than expected (84–87.5%) for patients with BM from ESCC/EAC (6, 11). However, due to the small sample size, the relatively high female-to-male ratio of 44.4% for patients with BM from SCEC in this study remains to be confirmed. The median age of the whole cohort was 59.5 years, which is similar to previous reports on ESCC and EAC (with the median age ranging from 60.7 to 65 years) (6, 12). The primary tumor was mostly located in the mid-thoracic esophagus (14/18, 77.8%). It has been reported that the rate of solitary BM from ESCC is 50–78.1% (6, 9). In the current study, nine cases developed solitary BM, accounting for 50% in the reported range for BM of ESCC, suggesting no association between the number of BM and the pathological subtype of EC.

Small-cell esophageal carcinoma is usually treated comprehensively as per the treatment guidelines for SCLC (13). Jeene et al. (14) reported that 58 patients with SCEC had an incidence rate of 12% for BM between 2000 and 2020. Six of the 58 patients received prophylactic cranial irradiation (PCI) treatment, and PCI treatment was not associated with the prognosis. From 2006 to 2018, we screened 270 patients with SCEC, none of whom received PCI. The incidence rate of BM from SCEC in this study is lower than that reported by Jeene et al. It is not clear if PCI treatment for SCEC is beneficial as the incidence of BM in SCEC is only 6.7% in this study and the PCI treatment was not associated with the prognosis in the study by Jeene et al. In this study, 13 patients received brain radiotherapy, accounting for 72.2% of the entire cohort. Thus, brain radiotherapy was the main treatment for BM from SCEC in practice. The median OS in the group of patients who received brain radiotherapy was 11.9 months, which is significantly higher than that of the patients who did not receive brain radiotherapy (*p* = 0.018). Song et al. (9) studied 73 patients with BM from ESCC. They found that the median OS of patients who received brain radiotherapy was significantly higher than that of patients who did not receive brain radiotherapy (7.13 vs. 3.4 months). Similarly, Domeki et al. (6) reported eight patients with BM from EC and demonstrated that brain radiotherapy could provide good local control, improve neurological symptoms, and achieve curative effect if the extracranial disease was well-controlled. These studies reveal that brain radiotherapy is still an indispensable treatment method and could improve the survival of patients with BM from EC, especially before effective systemic drugs are available to control intracranial metastases.

The present study showed that, although brain radiotherapy had significantly prolonged the median OS, the prognoses of patients were still poor, and all patients died within 2 years. This study suggests that the exploration of effective systematic treatment for these patients is necessary. At present, targeted therapy, anti-angiogenic therapy, and immunotherapy have been widely used for the treatment of lung cancer, malignant melanoma, and many other malignant tumors. However, there is a lack of effective targeted drugs for SCEC. Clinical studies of immunotherapy and anti-angiogenic therapy for SCEC are even less adequate. The DS-GPA grading standard (3), commonly used in patients with BM from the digestive tract tumor, was used in this study. The study showed that the median OS of patients with the DS-GPA score of 2.5–4 points was significantly higher than that of patients with scores of 0–2 points, which is consistent with the previous reports on BM from a malignant tumor of the digestive tract (11). However, this study used the DS-GPA score to evaluate the prognosis of patients with BM from SCEC. There were 22.2% of patients with extracranial metastases in this study. We failed to find a correlation between extracranial metastases and OS. Overall, the DS-GPA scores that include KPS, age, number of BM lesions, and the status of extracranial metastases may be more suitable for pretreatment evaluation of patients with SCEC and patients with BM.

The present study had some limitations. First, this was a retrospective and single-center study, leading to selection bias. Second, due to the small sample size, it is difficult to determine the optimal radiation dose and techniques. Third, given that the routine brain MRI screening was not undertaken for patients with SCEC in this institution, patients having the symptomatic disease were more likely to have taken brain MRI scans at earlier time points, and therefore, there may be an inherent bias in the OS as it represents the time from the diagnosis of BM to the time of death. Therefore, further prospective randomized research with larger samples is warranted. In addition, we are looking forward to the active emergence of more studies about targeted therapy, anti-angiogenic drugs, and immunotherapy for BM from SCEC, which can accelerate the development of treatment for patients with BM from SCEC.

In conclusion, the prognosis of BM from SCEC is very poor, the median OS is 7 months, and the 1-year survival rate is only 22.2%. The female-to-male ratio was relatively higher in patients with SCEC than in patients with ESCC and EAC. The DS-GPA score may be an important prognostic factor in patients with BM from SCEC. Brain radiotherapy could prolong the overall survival of patients with BM from SCEC. The findings have considerable practical implications and may be useful in future clinical trial design.

## Data Availability Statement

The raw data supporting the conclusions of this article will be made available by the authors, without undue reservation.

## Ethics Statement

The studies involving human participants were reviewed and approved by Ethics Committee of the Fourth Hospital of Hebei Medical University. The patients/participants provided their written informed consent to participate in this study.

## Author Contributions

JW: conceived and designed the study. WJ, LX, XZ, MH, and MZ: performed the study and analyzed the data. WJ and CL: wrote the paper. JW and CL: supervised the entire study and review the final paper. All authors listed have made a substantial, direct and intellectual contribution to the work, and approved it for publication.

## Conflict of Interest

The authors declare that the research was conducted in the absence of any commercial or financial relationships that could be construed as a potential conflict of interest.
